# Screening the visual system homeobox 1 gene in keratoconus and posterior polymorphous dystrophy cohorts identifies a novel variant

**Published:** 2013-04-11

**Authors:** Andrea L. Vincent, Charlotte Jordan, Leo Sheck, Rachel Niederer, Dipika V. Patel, Charles N.J. McGhee

**Affiliations:** 1 Department of Ophthalmology, New Zealand National Eye Centre, Faculty of Medical and Health Science, University of Auckland, New Zealand; 2 Ophthalmology Department, Greenlane Clinical Centre, Auckland District Health Board, Auckland, New Zealand

## Abstract

**Purpose:**

Mutations in the visual system homeobox 1 (*VSX1*) gene have been described at a low frequency in keratoconus and posterior polymorphous corneal dystrophy (PPCD). The putative role is controversial for several reasons, including a lack of mutations detected in other population cohorts. This study aims to determine whether *VSX1* contributes to the genetic pathogenesis of keratoconus and PPCD in a New Zealand population, and includes analysis of a Polynesian population.

**Methods:**

Recruitment of patients with keratoconus and PPCD, comprehensive clinical examination including corneal topography and pachymetry, and collection of biologic samples for DNA extraction were undertaken. Mutational analysis of *VSX1* (exons 1–7) with PCR and sequencing with bioinformatic assessment of variants was performed. Probable pathogenic variants were screened for in a control population using high-resolution melting analysis.

**Results:**

Forty-seven patients with keratoconus, including 15 familial cases, and ten unrelated patients with PPCD were recruited. Two pathogenic changes were detected; a novel change c.173C>T (p.Pro58Leu) was found in a patient with PPCD, predicted to be pathogenic, and not seen in 200 ethnically matched control alleles. The previously reported c.731A>G (p.His244Arg) was detected in a patient with sporadic keratoconus, and not present in the controls. No family members were available for segregation analysis.

**Conclusions:**

This study reports the presence of pathogenic mutations in *VSX1* in PPCD and keratoconus, including a novel disease-causing variant. The affected numbers are small, but given the growing body of evidence of pathogenic segregating changes in *VSX1* in disease cohorts, the expression in keratocytes as part of wound healing, and the documented association of PPCD and keratoconus, it seems likely that the role of *VSX1* as a genetic factor contributing to disease is real.

## Introduction

The visual system homeobox 1 (*VSX1*) gene is a member of the “paired-like” homeodomain transcription factors. This family plays a role in craniofacial and ocular development. Human *VSX1* has been mapped to 20p11.2. It was initially reported as containing five exons and approximately 6.2 kb in size [1] with an additional two exons characterized [2,3] that encode isoforms of the *VSX1* transcript. The expression of VSX1 has been detected in embryonic craniofacial tissues, adult retinas, and adult corneas [1,4]. Mutations in *VSX1* were reported associated with craniofacial abnormalities, empty sella tunica, and abnormal retinal cells [5], but more frequently and controversially with several corneal dystrophies and ectasias, specifically keratoconus and posterior polymorphous corneal dystrophy (PPCD).

*VSX1* was initially implicated in the pathogenesis of PPCD in 2002 [6]. PPCD is a frequently asymmetric autosomal dominant corneal dystrophy with characteristic involvement of Descemet’s membrane and the endothelium. In three families, mutations of *VSX1* were reported to segregate with the disease [6,7], but this was not replicated in other studies [8,9].

PPCD is genotypically heterogeneous: The largest percentage of PPCD (approximately one third) is associated with mutations in *ZEB1*, at the PPCD3 locus [10]. Haploinsufficiency results in ectopic collagen type IV, alpha 3 (COL4A3) expression in the cornea. A mutation in another gene, *COL8A2*, was reported in one family with PPCD [11] as well as in Fuchs endothelial corneal dystrophy. No further PPCD reports have been described with *COL8A2* mutations suggesting this association is tenuous or of a low frequency.

The relationship between keratoconus and *VSX1* was first reported in the study by Heon et al. [6]. The phenotypic heterogeneity of *VSX1* with involvement in keratoconus and PPCD is feasible as the disorders share a potential common mode of involvement of the posterior surface of the cornea, specifically Descemet’s membrane. The association of PPCD with keratoconus is also well documented with many cases described cooccurring in the same cornea [12-16].

Several mutations linked to keratoconus have since been identified [2,6,17-20]. The role of *VSX1* in the pathogenesis of keratoconus has also been controversial. Several other studies have failed to identify an association between *VSX1* variants/polymorphisms and keratoconus [21-24]. These contradictory results may be partly attributed to the low frequency of changes, ethnic variation, and the mounting evidence that keratoconus is likely a multifactorial and polygenic disease [25].

The variety of genetic techniques used to identify keratoconus genes has included family-based linkage studies, identity by descent, genome-wide scans, and genome-wide association studies. These approaches have identified a host of genetic loci and candidate genes [26], which appear to account for only a small number of those affected. Recently, association of keratoconus with the hepatocyte growth factor, *HGF* [27], and the microRNA *MIR184* [28] genes was identified.

Although anecdotally it is widely believed that keratoconus is more prevalent and aggressive in New Zealand, especially in the Maori and Pacific Island population, exact figures are not available [29,30]. However, keratoconus is the leading indication for corneal transplantation in adults and children in New Zealand [31,32]. It is plausible that a genetic factor is responsible for the ethnic predisposition of keratoconus in New Zealand. This study examines whether *VSX1* plays a role in the pathogenesis of keratoconus and PPCD in a New Zealand population.

## Methods

### Patient recruitment

Patients were recruited from the Department of Ophthalmology, Greenlane Clinical Centre, Auckland District Health Board with a clinical diagnosis of keratoconus or PPCD, and reviewed at the University Clinic, Department of Ophthalmology, University of Auckland. The protocol of this study adhered to the tenets of the Declaration of Helsinki with Institutional Ethics and Maori Research Review Board approval (Ministry of Health NTX/06/12/161 and ADHB A+3657).

### Clinical

Forty-seven healthy subjects (demographics provided in Table 1) underwent extensive clinical examination, including Snellen visual acuity, autorefraction, corneal topography, and pachymetry using a combined Placido/slit-scanning elevation tomography system (Orbscan II; Bausch & Lomb Surgical, Rochester, NY) and/or Pentacam Schiempflug analysis (Oculus, Wetzlar, Germany), slit-lamp examination and photography, and laser scanning in vivo confocal microscopy (IVCM) using the HRTII (Heidelberg Retina Tomograph II, Rostock Corneal Module [RCM]; Heidelberg Engineering GmbH, Heidelberg, Germany).

**Table 1:** Demographics of cohort studied.

Disease	Number	Age(years)	Sex	Familial	Caucasian	Polynesian	Indian
Keratoconus	47	41.5 (15 - 83)	20F:27M	15	17	26	4
PPCD	10	50.77 (16 - 86)	5F:5M	1	8	2	
Total	57	41.42	28F:34M	16	25	28	4

The table shows the distribution of Age in years, female:male ratio, familial cases and ethnicity as well as disease type of the cohorts investigated. F=female, M=male.

### DNA collection

After informed consent was received, biologic samples (10 mls of peripheral venous blood) were obtained through venesection with an ethylenediamine tetraacetic acid (EDTA)-coated Vacutainer (Greiner bio-one, Austria) and stored in a 4 °C refrigerator, or saliva specimen) were collected for DNA extraction using the salt extraction method from blood [33], and according to the manufacturer’s instructions for saliva kits (Oragene, DNAGenotek, Ottawa, Canada). For controls, DNA samples were collected from randomly selected and ethnically matched individuals attending the Ophthalmology Department who did not exhibit any clinical evidence of corneal abnormality in terms of appearance or topographic parameters.

### Mutational analysis of genes

DNA samples were screened for mutations in all coding exons of *VSX1* (NT_011387.8), including intron–exon boundaries (which included exons 6 and 7). Further details of primers and PCR conditions provided in Appendix 1. Following column purification with the HighPure PCR purification kit (Roche Diagnostic, Mannheim, Germany), the product was sequenced directly according to the protocols accompanying the ABI BigDye Terminator kit v3.1 (Applied Biosystems Inc., Foster City, CA). Bidirectional sequencing of amplicons was undertaken on an ABI 3700 prism genetic analyzer (Applied Biosystems). Nucleotide sequences were compared with the published *VSX1* sequence and polymorphic variation data in electronic databases to determine pathogenicity. For any uncharacterized or equivocal sequence change, a control population was screened with high-resolution melting analysis (HRMA).

To determine the frequency of the *VSX1* variants c.173C>T (p.Pro58Leu), and c.731A>G (p.His244Arg), 100 ethnically matched control individuals (200 alleles) were screened. Screening for the detected *VSX1* sequence variants used HRMA on the RotorGene6000 (Corbett Life Sciences, San Francisco, CA), using the High Resolution Melting Master kit (Roche Diagnostic). Further details of primers and conditions are provided in Appendix 1. Each reaction included a positive and negative control based on the sequencing confirmation. Any sample on the melt curve that produced an equivocal reading was subject to further PCR and sequencing to confirm or exclude the presence of the sequence variation.

For the sequence variants, homology and predicted destruction or creation of exonic splicing enhancers, or effects on splicing, were evaluated using various publicly available software. PolyPhen2 and Sorting Intolerant From Tolerant (SIFT) analysis was used to predict the impact of the c.173C>T (p.Pro58Leu) and c.731A>G (p.His244Arg) missense variants on protein structure and function. Potential pathogenicity was based on positive family segregation, an allele frequency of <1/100 control chromosomes, homology, and bioinformatic prediction of biologic significance.

PolyPhen2 analyzes an amino acid variant at the structural level, to assess any functional implication of an amino acid change [34].

SIFT is another sequence homology-based tool, which works on the philosophy that conservation of proteins throughout evolution is tightly correlated with the function [35]. Therefore, SIFT aims to predict the phenotypic effect any amino acid substitution will have, based on whether it is tolerated.

## Results

**Figure 1:**
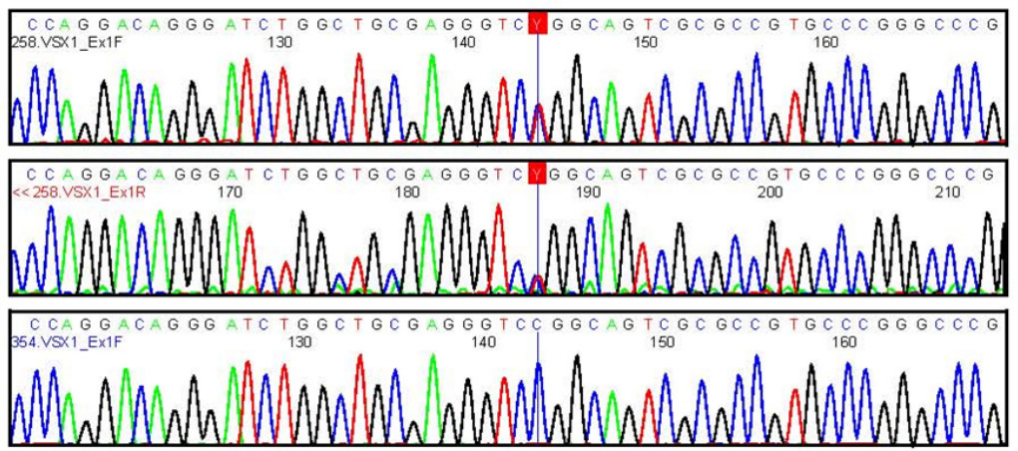
The electropherogram of the proband with posterior polymorphous corneal dystrophy shows the heterozygous sequence variant c.173C>T, which results in the protein change p.Pro58Leu. The top panel shows the forward sequence, the middle panel shows the reverse sequence (reverse complement shown) and the bottom panel in a control shows the wild type sequence c.173 C/C.

**Figure 2:**
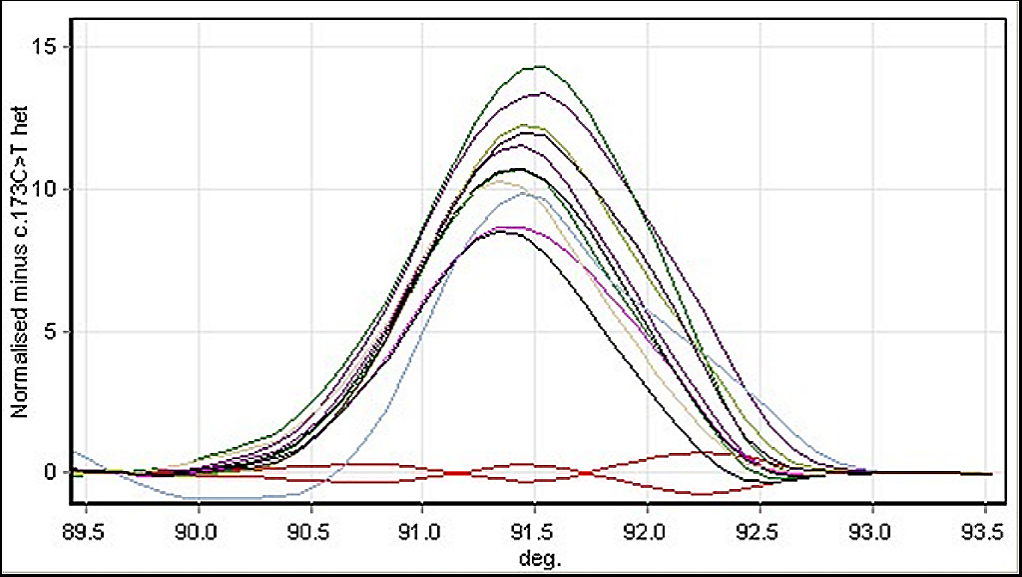
High-resolution melting analysis normalized difference graph of screening in the control population (200 alleles) for the *VSX1* c.173C>T variant. The melt profiles shown in red at the bottom are duplicate samples of the positive control (affected c.173C>T heterozygous) samples. All other samples with no variants are represented by the bell-shaped melting curves.

Forty-seven patients with keratoconus, including 15 familial cases, and ten patients with PPCD were included; their demographic features summarized in Table 1. One novel variation in exon 1 in the proline-rich area (44–127) c.173C>T (p.Pro58Leu; Figure 1) was found in a patient with PPCD. The variation was not present in 200 control alleles (Figure 2 HRMA analysis), and homology showed the variation is highly conserved. Protein prediction with PolyPhen2 suggests this mutation is “probably damaging” with a position-specific independent count (PSIC) score of 2.299 and a SIFT score of 0.04 also labeled as “damaging.” Population screening has detected this allele in 3/8,075 alleles (Exome Variant Server [EVS], accessed September 20, 2012) with a minor allele frequency of 0.0371%, but the allele is not listed in the 1000 Genomes Database (accessed September 20, 2012).

**Figure 3:**
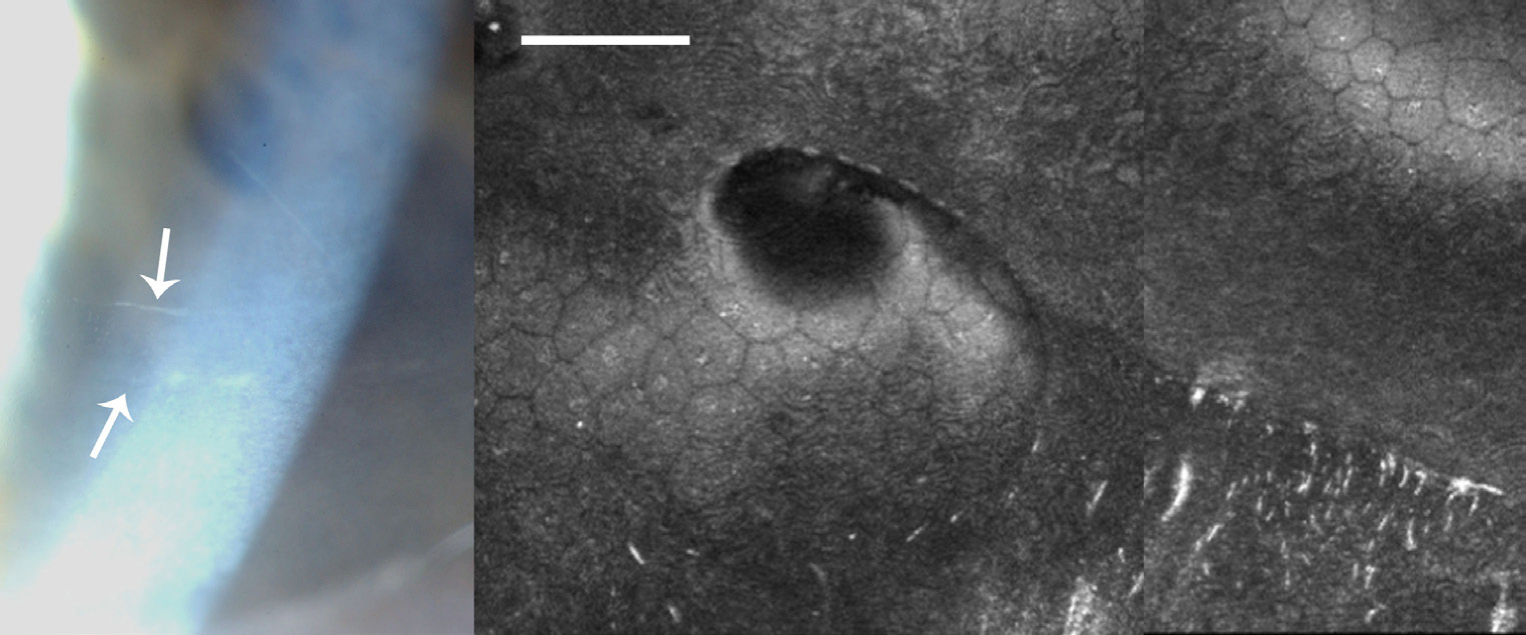
Clinical images of Case 1. **A**: A slit-lamp photograph of Case 1 showing a posterior polymorphous dystrophy band lesion (arrows). **B**: In vivo confocal microscopy in this patient shows undulation of Descemet’s membrane and the endothelial surface, with needle-shaped hyper-reflectivity at the level of Descemet’s membrane. (Scale bar=100 μm)

This female subject was a 57-year-old Caucasian, with no family history of PPCD or keratoconus. She had a history of open angle glaucoma and had previously undergone a left trabeculectomy. Visual acuity was 6/5 (right eye) and 6/6 (left eye). Corneal tomography (Orbscan II) demonstrated minor asymmetry with minimal inferior steepening; however, all parameters were within normal limits regarding features of keratoconus: right simulated keratometry 43.8 diopter (D)/42.7 D with 1.1 D of astigmatism at 95° and thinnest pachymetry 519 μm; left simulated keratometry 45.9 D/44.7 D with 1.2 D of astigmatism at 97° and thinnest pachymetry 524 μm. The right eye demonstrated no features of PPCD with a mean endothelial cell density of 2,351 cell/mm^2^, whereas the left cornea, although entirely clear, demonstrated classical “vesicular” PPCD features of multiple endothelial vesicles (Figure 3A) and curvilinear ridges with a mean endothelial cell density of 1,323 cells/mm^2^ (HRTII; Figure 3B). The anterior segments of both eyes were otherwise normal.

**Figure 4:**
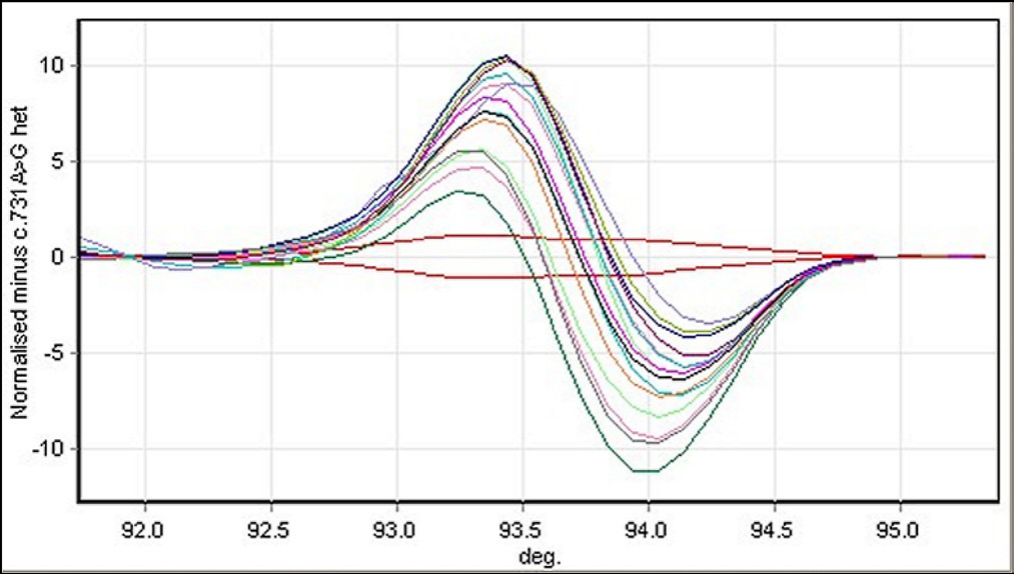
High-resolution melting analysis normalized difference graph of screening in the control population (200 alleles) for the visual system homeobox 1 gene c.731A>G, p.His244Arg heterozygous variant. The duplicate red lines running parallel to the x-axis at zero are the positive control (affected) samples, while the samples with no variants are represented by the sine-shaped melting curves.

The previously described c.731A>G (p.His244Arg) was detected in one patient with sporadic keratoconus. This variant lies within the conserved Chx10/Vsx-1 and ceh-10 (CVC) domain (224–277). The variant was not detected in the 200 control alleles (Figure 4), and protein prediction with PolyPhen2 suggests that this variant is probably damaging, with a PSIC score of 2.532. SIFT analysis also calls this change “deleterious.” Population screening has detected this allele in 27/12,059 alleles (Exome Variant Server, accessed September 20, 2012).

**Figure 5:**
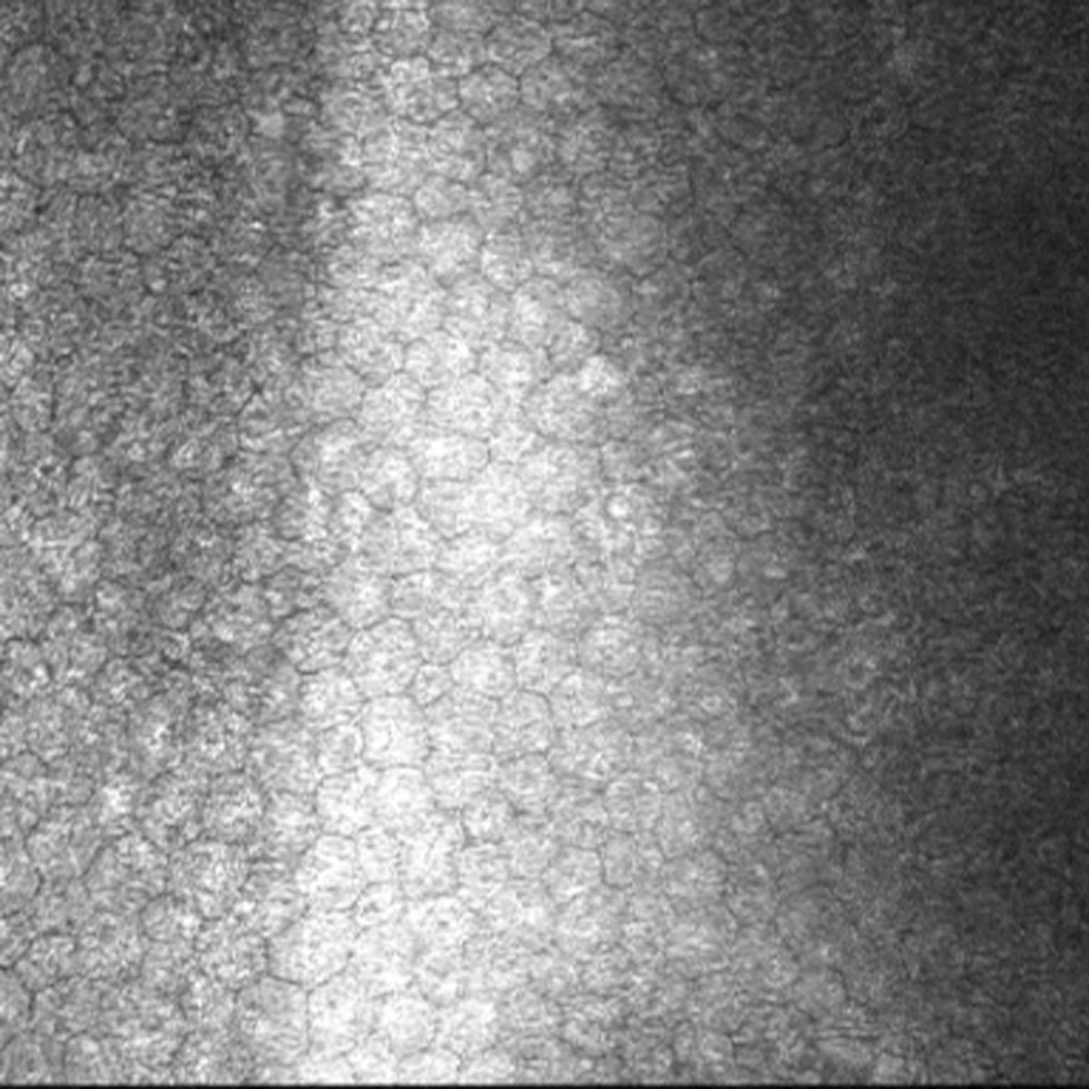
In vivo confocal microscopy in this patient with keratoconus, heterozygous for the visual system homeobox 1 p.His244Arg variant shows a healthy endothelium. (Scale bar=100 μm)

This male subject was Caucasian, 42 years of age with asymmetric keratoconus, right worse than left, and corrected visual acuities with contact lenses were 6/9 (right eye) and 6/6 (left eye). Orbscan II computerized keratometry-confirmed bilateral keratoconus. A right asymmetric bowtie appearance with inferior steepening was associated with simulated keratometry of 53.3 D/46.4 D with 6.9 D of astigmatism at 100° and thinnest pachymetry of 425 μm on Pentacam analysis. Left Orbscan tomography highlighted atypical inferior steepening of early keratoconus with simulated keratometry 44.6 D/43.2 D with 1.4 D of astigmatism at 119° and thinnest pachymetry of 467 μm on Pentacam Scheimpflug analysis. No evidence of PPCD was identified by an experienced corneal subspecialist, and laser scanning in vivo confocal microscopy of the cornea (HRTII) confirmed normal endothelial morphology (Figure 5). Unfortunately, no family members were available for segregation analysis.

## Discussion

This study investigated *VSX1* changes in all seven exons in a population with PPCD and keratoconus, with a significant Polynesian ethnic proportion. Two likely pathogenic changes were detected in two Caucasian individuals. The c.173C>T occurs in the proline-rich domain causing the amino acid change p.Pro58Leu, which is highly conserved, not seen in our control population, and predicted to be pathogenic.

The second observed change c.731A>G, p.His244Arg was identified in a Caucasian patient with keratoconus and has previously been described in several papers [6,24]. Heon et al. [6] observed this change in 1/63 patients with keratoconus and segregated with the disease, but also observed it in 2/277 controls. Tang screened a case control panel (70 controls and 77 patients with sporadic keratoconus) but did not observe His244Arg in this panel [24]. That study also screened 444 individuals from 75 families: Two affected and one unaffected had the variant, although it is not clear from this paper if forme fruste keratoconus was included as a parameter for affectation status. The presence in a control population has led to doubts about its pathogenicity.

The role of *VSX1* in the pathogenesis of keratoconus and posterior polymorphous dystrophy has been debated in the literature since the gene was first described in 2002 [6]. Several factors contribute to this debate, but one point is the small number of cases of PPCD and keratoconus in which changes in *VSX1* are described, despite probable pathogenic changes being described since 2002 in multiple population cohorts. Other factors that have placed doubt upon the validity of the role of this gene in these disorders include changes in the gene not detected in other cohorts, non-segregation of variants, presence of supposed pathogenic alleles in the control population, and questionable corneal expression. Only one study to date has examined exons 6 and 7 [2].

Although *VSX1* expression was initially reported in the cornea [1], further studies clearly demonstrated expression only in the retina [36,37]. Indeed, *Vsx1* expression was not determined in the mouse cornea or in adult human corneas [6], and a mutant *Vsx1* mouse model had no corneal phenotype [38]. Subsequently, however, *VSX1* expression in keratocytes has been characterized in vitro and in vivo using real-time PCR, immunohistochemistry, and in situ hybridization [4]. Although not observed in resting or quiescent human keratocytes, in wounded corneas, or when cultured in serum to mimic wounded conditions, the keratocytes express *VSX1*, and this is associated with fibroblastic transformation [4]. These observations add strength to the hypothesis that VSX1 is involved in the wound healing response and thus may contribute to the underlying pathology in corneal disease. Interestingly, recent work in damaged and normal mouse corneas failed to demonstrate any Vsx1 expression, with the authors concluding there may be a species-specific role for *VSX1* [39].

In addition, the original PPCD1 locus (MIM# 122000) was mapped to a 30 cM pericentromeric locus on 20p11-q11 [40]. When the locus was refined in Czech families in 2005, *VSX1* sat outside this interval [9]. The PPCD1 locus was further reduced to 2.4 cM [41] and subsequently probed with Sanger and next-generation sequencing [41,42]. The underlying genetic cause within this locus appears to remain elusive. A recent publication further explored this locus demonstrating a founder haplotype in the Czech population, but no causative mutation was identified [43].

Regarding the role of *VSX1* in keratoconus, one of the most recent publications on this subject looked at an Italian cohort of 302 patients with keratoconus (the largest series published to date) and found probable pathogenic changes in *VSX1* in 3.2% of the affected population. In addition, the authors emphasized the possibility of variable expressivity and incompletely penetrant mutations, as well as the possibility the *VSX1* changes are a genetic predisposing factor in this multifactorial disease [44]. These concepts (variable expressivity, incomplete penetrance, and genetic risk alleles) are well documented and accepted in a host of other diseases affecting the eye such as neurofibromatosis type 1, retinoblastoma, and age-related macular degeneration. A study of Iranian patients with keratoconus also showed p.His244Arg segregating with disease in a two-generation pedigree; four affected were heterozygous whereas five unaffected were not, and it was not present in extensively phenotyped controls [45]. Analysis of the pedigrees demonstrated a 58% reduced penetrance in general among the Iranian families with keratoconus, which could explain Tang et al.’s finding [24]. Other recent studies also demonstrate segregation of other *VSX1* probable pathogenic changes with keratoconus [44,46].

A recent paper characterized the cornea in patients with PPCD showing the topographic parameters were significantly steeper, but with no clinical or topographical evidence of keratoconus [47]. In this paper, of the 18 individuals characterized, 14 were from six families, and eight patients were below the age of 14 years. Twin studies have demonstrated a genetic component to corneal curvature, and the younger members may be too young to assess whether they truly have keratoconus as the disease may not manifest until later teens. Alternatively, this group may have described a subgroup of individuals in whom PPCD and steep corneal curvature are inextricably genetically linked. This group of patients was also not genetically characterized. In another study of six patients with *ZEB1* mutations, three had steep corneas but no evidence of keratoconus [48].

A limitation of our study is the small number of patients, particularly in the PPCD cohort. In addition, family members were not available for those with mutations to prove or disprove segregation. As the greatest percentage of our patients with keratoconus were of a unique Polynesian ethnicity, this may suggest that *VSX1* does not play a causative role within this ethnic group.

This study confirms the presence of pathogenic mutations in PPCD and keratoconus. The affected numbers are small, but given the growing body of evidence of pathogenic segregating changes in *VSX1* in these cohorts, the expression in keratocytes as part of wound healing, and the documented association of PPCD and keratoconus, it seems likely that the role of *VSX1* is real, but not significant in terms of actual numbers. Genetic heterogeneity is almost the norm in a large number of complex eye conditions such as the retinal dystrophies and glaucomas, with these corneal disorders also demonstrating a similar complexity in genetic causation.

## Appendix 1. VSX1 Primers and conditions.

To access the data, click or select the words “Appendix 1.”
